# An epidermal cyst of the penis after distal hypospadias surgery: a case report

**DOI:** 10.1186/s13256-018-1930-6

**Published:** 2018-12-24

**Authors:** Yavuz Guler, Zeynep Tatar, Burak Ucpinar, Akif Erbin

**Affiliations:** 1 Department of Urology, Safa Hospital, Istanbul, Turkey; 2 Department of Pathology, Patomer Pathological Cytological Research Center, Istanbul, Turkey; 3 Department of Urology, Haseki Traning and Research Hospital, Istanbul, Turkey

**Keywords:** Distal hypospadias, Epidermoid cyst, Penile surgery, Urethral dehiscence

## Abstract

**Background:**

Epidermoid cyst is a benign tumor that can occur anywhere in the body but is rarely seen in the penis. Congenital and previous penile surgeries have been reported to be involved in the etiology of the disease, which is usually asymptomatic. Here we describe a case of a patient with a penile epidermoid cyst, which occurred in the circumcision line on the left side of his penis, and urethral dehiscence following hypospadias surgery.

**Case summary:**

A 3-year-old white boy who underwent primary distal hypospadias surgery 1.5 years ago presented with a slowly growing mass in the left ventrolateral portion of the penile circumcision line and urethral dehiscence. The histology of the excised mass revealed an epidermal inclusion cyst. Since then, he has remained healthy.

**Conclusions:**

Epidermal inclusion cyst as a complication of hypospadias surgery is a very rare situation. The diagnosis is made histologically and surgical excision is sufficient for treatment.

## Background

Epidermal inclusion cyst is a benign lesion that can develop in any part of the body. Although cutaneous epidermoid cyst is a common lesion, penile localization of these lesions is quite rare. In fact, only a few cases of penile epidermoid cysts have been reported in the literature and the vast majority of cases were congenital and/or idiopathic lesions diagnosed during childhood [1]. The etiology of congenital epidermoid cysts is unknown but it has been suggested that it may represent a monolayer teratoma of germ cell origin or abnormal embryologic closure of the median raphe [2]. In previously published articles, penile epidermoid cystic lesions have been reported as slow-growing, solitary, well-demarcated cystic lesions with a smooth and soft appearance and, when properly removed, they are not recurrent [3]. To the best of our knowledge, epidermoid cyst following distal hypospadias surgery is very rarely encountered. In this case presentation, we aimed to describe the diagnosis and management of this rare entity.

## Case presentation

A 3-year-old white boy who had undergone distal hypospadias surgery 1.5 years ago presented with a slowly growing mass on the left side of his penis and dehiscence of the urethra. His medical, social, environmental, and family history were otherwise unremarkable. His developmental milestones and psychosocial status were compliant with his percentile. His parents were non-consanguineous.

**Fig. 1:**
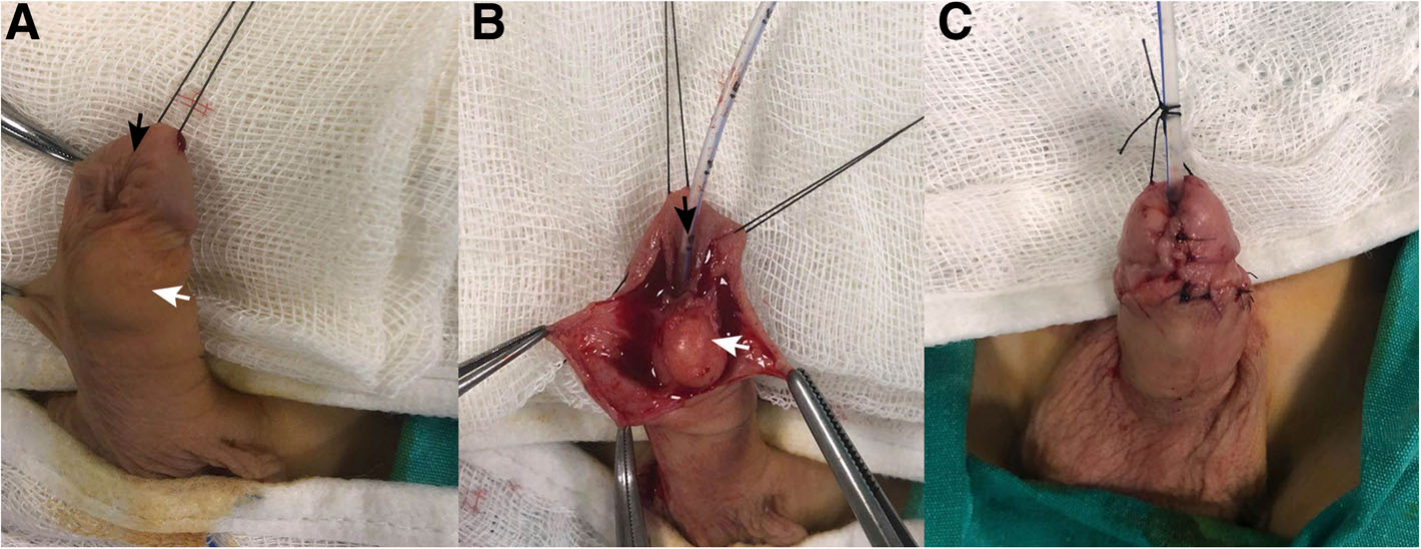
**a** *White arrow* shows the localization of the epidermal cyst and *dark arrow* shows urethral dehiscence. **b** *Dark arrow* shows the urethral dehiscence. **c** The appearance after cyst excision and tubularized incised plate urethroplasty surgery

**Fig. 2:**
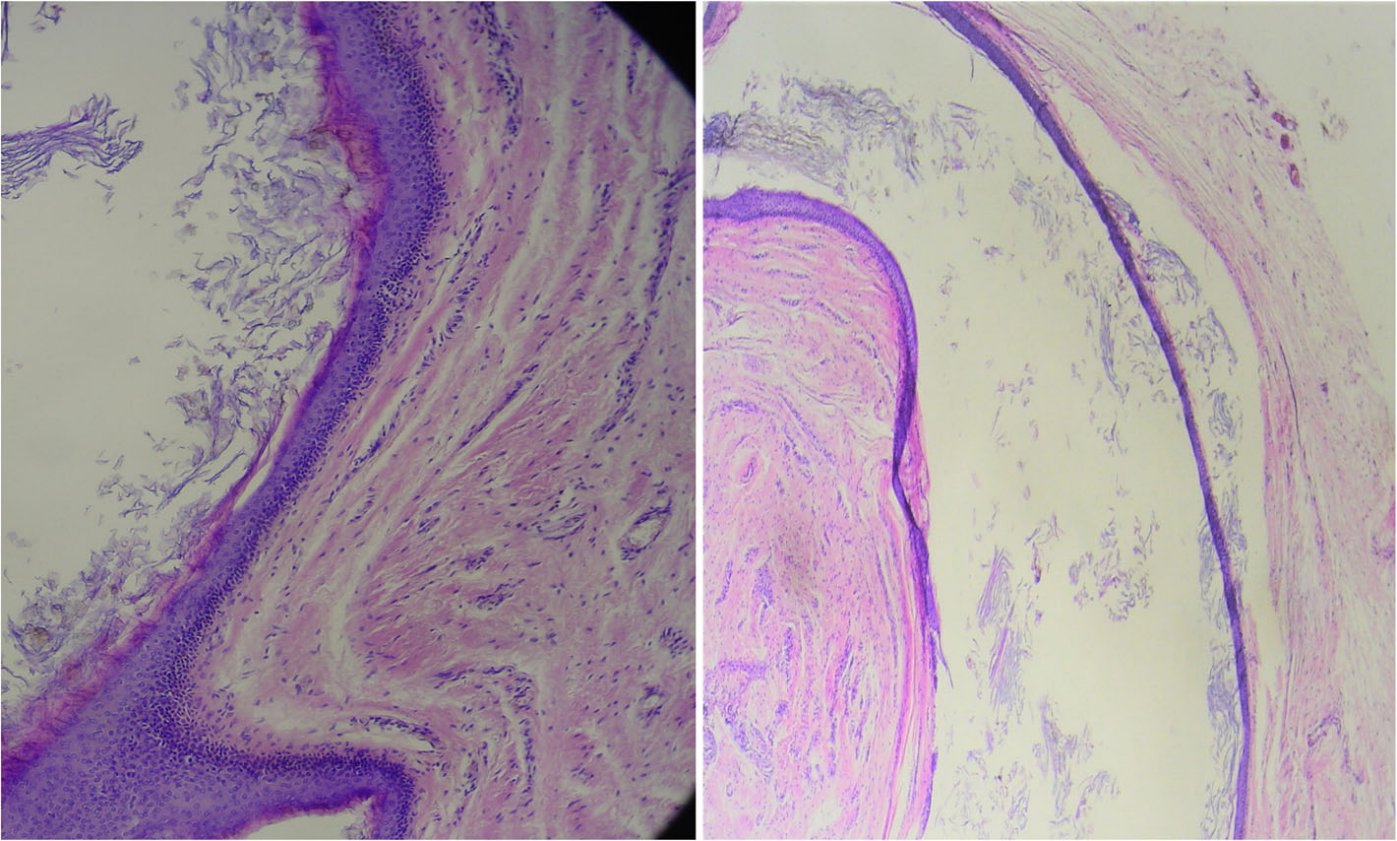
Pathological examination. In the lumen of the multilayered squamous epithelium-coated cystic formation, lamellated keratin material content is seen

On admission, his temperature was 36.2 °C, pulse was 96 beats/minute, and blood pressure was 85/54 mmHg. A physical examination revealed the distal urethral dehiscence and the pattern of urine flow was abnormal. The mass which was located in the ventral aspect and distal part of his penis was painless and hard in texture. The dimensions of the mass were 2 × 2.5 cm (Fig. 1). Bilateral testes were in his scrotum and normal sized according to the age. A neurological examination of the child was unremarkable. In laboratory analysis, total white blood cell (WBC) count was 6.9 10^3^/mm^3^, hemoglobin was 12.4 g/dL, alanine aminotransferase (ALT) was 14 u/l, aspartate aminotransferase (AST) was 18 u/l, and creatinine was 0.6 mg/dl. Urine analysis showed normal amounts of red cells with no suspicion of urinary tract infection. For treatment, tubularized incised plate urethroplasty (TIPU) and accompanying cyst excision were performed. In histopathological examination of the mass, it was determined that the wall of the epidermoid cyst was composed of multilayered squamous epithelium which was keratinized toward the lumen (Fig. 2). His post-operative course was uneventful and no urethral fistula occurred during 6-month follow-up. His urine flow was straight and the cosmetic outcome of the surgery was satisfactory.

## Discussion

In this case report we have presented an epidermal penile cyst, following hypospadias surgery in a pediatric patient. We have discussed the details of diagnosis and management of this rare entity.

Epidermoid cysts of the penis are mainly encountered in children and they are very rare among elderly patients [3, 4]. Epidermoid cysts are common benign tumors that may arise from the infundibular portion of the hair follicles spontaneously or subsequent to trauma [5]. These cysts can occur in various parts of the body but are found most commonly in the vulva, especially in populations where female circumcision is practiced [1, 6].

Penile epidermal cysts are broadly classified into congenital cysts and secondary cysts caused by trauma or surgery. Congenital cysts may arise from the accumulation of the epidermal secretions during embryonic life, whereas secondary cysts may arise from traumatic implantation of epithelial elements or the occlusion of the pilosebaceous unit. Congenital cysts, such as median raphe cysts, can arise from an abnormal embryological closure defect of the median raphe [1, 5] or a monolayer germ cell teratoma, as proposed for intratesticular epidermoid cysts [6]. Median raphe cysts can occur anywhere along the genitoperineal raphe from the urethral meatus to the anus. Most researchers believe that median raphe cysts are the sequelae of an error in the embryologic development of the male genitalia [6]. During normal development, the paired genital folds are positioned at the base of the genital tubercle, and gradually envelop the urethral plate and merge in the midline to create the bulbar and pendulous segments of the urethra. The glandular urethra is created by the coring action of an ectodermal ingrowth [6].

Secondary cysts can occur because of occluded pilosebaceous unit or traumatic/mechanical implantations involving the implantation of epithelial elements and obstructed eccrine ducts [4–8]. As in our case, traumatic secondary epidermoid cysts are known to occur after circumcision, hypospadias surgeries, and other penile surgeries (that is, penile enlargement surgery) [9, 10].

In general, penile epidermal cysts are asymptomatic, unless they are complicated by an infection or forceful coitus [7]. Most of the patients may remain asymptomatic during childhood and become symptomatic during adolescence or adulthood. Common symptoms are pain (due to infection or trauma), difficulty during micturition, hematuria, hematospermia, and difficulty in having sexual intercourse [6].

Penile epidermal cysts can be diagnosed by careful physical examination and radiological evaluation, including ultrasonography and computed tomography. Magnetic resonance imaging may be useful in cases where there is a suspicion of extension into the pelvis, although such cases are rare and there are a few cases in the literature [6, 7]. In cases of suspected extension, magnetic resonance imaging is useful for depicting the anatomical boundaries of the lesion [11]. In our case, radiological imaging was unnecessary because the boundary of our patient’s cyst was well demarcated and palpable. Major cysts in the root of the penis can be mistakenly diagnosed as hydrocele. In such cases, it is important to perform a physical examination and scrotal ultrasonography to exclude any scrotal malformation [5].

Although they occur in various sizes and lengths, penile epidermoid cysts are usually small, soft, freely movable, and solitary masses which are rarely multifocal. Differential diagnoses include dermoid cyst, teratoma, urethral diverticulum, glomus tumor, pilonidal cyst, epidermal inclusion cyst, and steatocystoma [3, 6, 11]. The skin and its appendages are present in dermoid cysts and derivatives of other germ cells are present in teratomas [5]. In addition, an evaluation of the presence of any urethral diverticula and urethrocutaneous fistula is important. These pathologies can usually be ruled out by both physical examination and the conditions evident upon voiding. When the diagnosis remains questionable, a voiding cystourethrogram should be obtained [6].

To the best of our knowledge, no cases of malignancy associated with penile cystic disease have been reported previously. Indications for the treatment of penile cysts are secondary cystic infections, pain during sexual intercourse, cosmetic complaints, or obstruction of the urinary tract [2]. In our case, a hypospadias surgery had to be performed and no extra indication was needed for cyst removal. Simple complete excision followed by primary closure has generally been regarded as the best treatment procedure. Aspiration and simple drainage may carry a risk of recurrence. It has been reported that re-excision was required when residual tissue was left after treatment [5]. In cases where no malignancy is evident, simple observation is adequate for follow-up. Surgical excision of penile epidermal cysts is the treatment of choice [5–8]. The resection should be completed without leaving any epithelium to prevent recurrence. Although no cases of malignancy arising in the wall of an epidermal cyst of the penis have previously been reported, patients with epidermal cysts should be followed up after complete cyst removal.

## Conclusions

Penile epidermoid cysts following hypospadias surgery is a very rare complication of this surgery. Epidermoid cysts should be kept in mind in cases of cystic, slowly growing penile lesions. Treatment of this pathology includes surgical resection and if concomitant urethral dehiscence is present, simultaneous repair can be considered.

## Abbreviations

ALT: Alanine aminotransferase
AST: Aspartate aminotransferase
TIPU: Tubularized incised plate urethroplasty
WBC: White blood cell
